# Baihe Jizihuang Tang Ameliorates Chronic Unpredictable Mild Stress-Induced Depression-Like Behavior: Integrating Network Pharmacology and Brain-Gut Axis Evaluation

**DOI:** 10.1155/2021/5554363

**Published:** 2021-08-24

**Authors:** Jian-ping Zhu, Hua-ying Wu, Yuan Zi, Xin-bin Xia, Meng-zhou Xie, Zhi-ying Yuan

**Affiliations:** ^1^Hunan University of Chinese Medicine, Changsha 410208, China; ^2^School of Medicine, Hunan Normal University, Changsha 410013, China; ^3^Hengyang Hospital of Traditional Chinese Medicine, Hengyang 421001, China; ^4^Key Laboratory of TCM Heart and Lung Syndrome Differentiation and Medicated Diet and Dietotherapy, Changsha 410208, China

## Abstract

Baihe Jizihuang Tang (BHT) is a traditional Chinese medicine (TCM) prescription, which can also be used as a nutritional food with medicinal value. Herein, we aimed to clarify the antidepressive effects and molecular mechanism of BHT. Network pharmacological analysis; chronic unpredictable mild stress (CUMS) rat model assessment; behavioral tests; analysis of hippocampal neurotransmitter levels, hippocampal pathological structure, and hypothalamic-pituitary-adrenal (HPA) axis; western blot analysis; 16s RNA sequencing; ultraperformance liquid chromatography (UPLC)/mass spectrometry (MS); and high-performance liquid chromatography (HPLC)/ultraviolet (UV) analysis were used. We found 8 potentially active components and 12 targets from the database. KEGG analysis suggested that BHT significantly affected BDNF/tyrosine receptor kinase B levels, glutamate binding, synaptic transmission based on neurotransmitter signal, and the response to glucocorticoid signaling pathways. Consistently, 7 chemical components were identified using UPLC/quadrupole time-of-flight/MS; among them, regalosides A, B, C, and E were unique components of lily of TCM, and their content in BHT was significantly different: regaloside A > B > E > C. BHT could nourish hippocampal neurons, affect neurotransmitter metabolism, reduce HPA axis hyperactivity, improve deficits in hippocampal tissue structure, and change depressive behavior. Moreover, BHT regulated BDNF expression in the hippocampus and improved intestinal flora deficits in CUMS rats by changing the content of *Bifidobacterium*, *Rothia*, *Glutamicibacter*, and *Lactobacillus* at the genus level. Collectively, BHT attenuated CUMS-induced depression-like behavior by regulating BDNF and intestinal flora disorder through the brain-gut axis. Therefore, including BHT in the medication list may constitute a potential strategy for preventing depression.

## 1. Introduction

Depression is a chronic mood disorder involving a complex pathophysiological process with multiple mechanisms. In 2017, the World Health Organization (WHO) announced that the global prevalence of depression had risen to 4.4%, with 322 million patients, and that depression was associated with a lifetime prevalence rate of about 16% [1]. Without timely detection and effective treatment, depression not only seriously affects the patient's family and social functioning but also may even lead to suicide [2].

Emerging evidence suggests that the hypothalamic–pituitary–adrenal (HPA) axis, neurotransmitters, intestinal flora, and brain-derived neurotrophic factor (BDNF) are involved in the pathology of depression. As a mediator of stress responses of the neuroendocrine system, the HPA axis is important for the multiple and complex interactions between the endocrine and immune systems [3]. Long-term stress increases HPA axis activity, leading to excessive secretion of adrenocorticotropic hormone (ACTH) and upregulation of inflammatory factors, which enter the brain through the blood-brain barrier causing neurotoxicity, thereby accelerating hippocampal injury and inducing depression [4]. Studies have shown significantly increased plasma levels of cortisol (CORT), adrenocorticotropic hormone (ACTH), and corticotropin-releasing hormone (CRH) in a rat depression model, as well as significantly increased expression levels of interleukin (IL) 1*β* and IL-6 mRNAs in the hippocampus but reduced 5-hydroxytryptamine (5-HT) levels [5, 6]. Neurotransmitter disorders are a prominent feature of depression, and intestinal flora can affect their production by regulating the function of intestinal mucosal epithelial cells [7]. American neurobehavioral geneticists proposed that intestinal microbe disorders can affect the function of the HPA axis, causing immune system dysfunction and triggering inflammatory responses [8]. Studies have shown higher levels of *Platella* and lower levels of *Bifidobacterium*, *Bacteroides*, and *Lactobacillus*, at the genus level, in the feces of depressed patients than in those of healthy individuals [9].

The neurotrophic factor, BDNF, is widely distributed in the central nervous system of mammals, and the BDNF/TrkB signaling pathway plays a key role in the differentiation, proliferation, nutrition, and maturation of various neuronal types. Animal experiments have confirmed that the expression of BDNF in the hippocampus is downregulated in chronic and unpredictable stress-induced depression [10–13]; bacteria that confer a health benefit, such as *Lactobacillus casei* and *Bifidobacterium* can improve depression-like behavior in rats with chronic unpredictable mild stress-induced depression through the BDNF/TrkB signal pathway [14], which has become a research hotspot in the field of depression in recent years.

Baihe Jizihuang Tang (BHT), also called Baihe Jizi decoction, recorded in the traditional Chinese medicine (TCM) as “synopsis of the Golden Chamber,” is widely used to treat nervous system disorders caused by “Baihe disease” (TCM disease name, related to mental disorders). The prescription contains two herbs, lily (mainly from the dry bulb of *Lilium lancifolium* Thunb.) and hen egg yolk, which can also be used as food. Recent studies have shown that lily contains multiple chemicals, including steroidal saponins, sterols, phenolic glycerides, flavonoids, phenylpropanol, alkaloids, and polysaccharides [15, 16]. Hen egg yolk is rich in protein, phospholipids, minerals, and vitamins [17, 18]. BHT is the only medicinal diet prescription in the treatment of “Baihe disease,” but its antidepressant mechanism has not been determined. Other than our fecal metabonomic research on BHT antidepressive action [19], there is no report on the antidepressant mechanism of this medicinal diet prescription. Therefore, it is of great innovative value to carry out research on the antidepressant effect of BHT.

**Figure 1:**
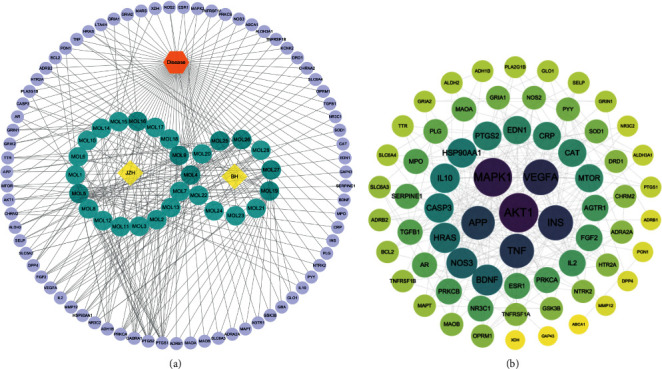
(a) Network of “drug-compound-target” of BHT and (b) target interaction network of high confidence of BHT of antidepressant effect.

**Figure 2:**
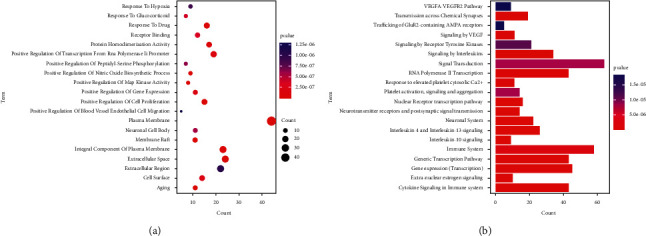
(a) Bubble plot of bioaccumulation analysis of GO and (b) bubble plot of Reactome path analysis.

**Figure 3:**
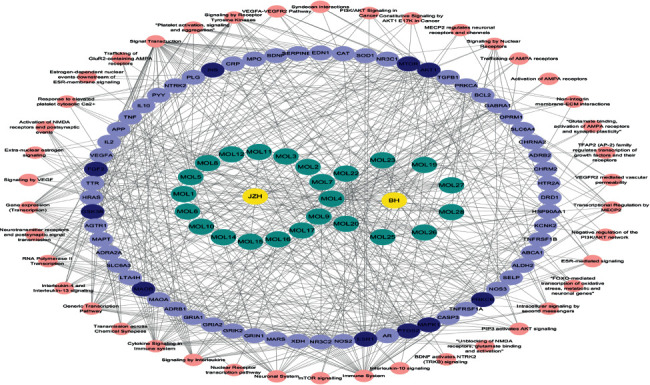
“Drug-compound-target pathway” network of BHT of antidepressant effect.

In this study, integration of network pharmacology and molecular mechanism evaluation were used for the first time to study the antidepressant mechanism of BHT. Network pharmacology analysis (Figures 1–3 ) indicated that BHT mainly targets the BDNF/TrkB signaling pathway, glutamate binding, synaptic transmission based on the neurotransmitter signal, and responses to glucocorticoids, thus mediating the antidepressive effects of components such as *β*-sitosterol, oleic acid, stigmasterol, and regalosides E, A, and B.

In the verification study, we analyzed the components by ultra-performance liquid chromatography (UPLC) mass spectrometry (MS)/high-performance liquid chromatography (HPLC) and evaluated the molecular mechanisms through animal experiments. First, the antidepressant effect of BHT was evaluated with behavioral tests used to assess depression-like behavior, the sucrose preference test (SPT), and the open-field test (OFT). Second, the related indexes of HPA axis activity, neurotransmitter content, hippocampal histopathology, BDNF/TrkB protein expression level, and intestinal differential microflora were integrated and analyzed to explore the brain-gut axis mechanism.

## 2. Materials and Methods

### 2.1. Network Pharmacological Method

Based on the literature [20] and TCMSP (http://tcmspw.com/index.php), the chemical components of BHT were searched, and compounds lacking the target prediction and including repeated data were removed. The active compounds of lily and hen egg yolk in BHT, as well as their protein targets, were obtained using the database SwissADME to screen for active compounds with better druggability. Moreover, the compounds of the chemical components of BHT were collected and screened using the SwissADME database. Target prediction and calibration of the active ingredients using TCMSP platform, SwissTargetPrediction database (http://www.swisstargetprediction.ch/), SEA database (http://sea.bkslab.org/), and STRING database were performed.

In the GeneCards database (http://www.genecards.org/), CTD database (http://ctdbase.org/), DisGeNET database (https://www.disgenet.org/), and OMIM database (http://www.omim.org/), depression-related genes were retrieved. These disease-related genes were selected and intersected with potential targets of lily and hen egg yolk to identify potential targets of BHT in the treatment of depression.

The selected active compounds and their target correspondence in lily and hen egg yolk were introduced into the Cytoscape V3.7.2 software (http://www.cytoscape.org/) to draw the “compound target disease” network and carry out topology analysis using this network. According to the degree of betweenness centrality (BC) and closeness centrality (CC), the key effective components of lily and hen egg yolk in the treatment of depression were selected and analyzed. Potential targets were uploaded to the string database, and network data with confidence ≥0.7 were imported into the Cytoscape software to draw the interaction network and carry out topology analysis and to select the core targets of BHT in the treatment of depression according to BC, CC, and degree value analysis.

Gene ontology (GO) biological process enrichment analysis was conducted for the predicted therapeutic targets using the DAVID (https://david.ncifcrf.gov/). Pathway analysis was performed using the Reactome database (https://reactome.org/), and a threshold of *P* < 0.05 was set. Pathways with *P* < 0.01 and false discovery rate <0.01 were selected to draw the “compound-target-pathway” network diagram in Cytoscape.

### 2.2. Apparatus and Reagents

The following instruments were used: Agilent UPLC-6410 QQQ/MS instrument (Agilent Technology Co., Ltd., USA), Waters ACQUITY UPLC/Xevo G2 QTOF/MS (Waters Technology Co., Ltd., USA), Shimadzu LC-10A HPLC (Shimadzu company, Japan), DH-250 electric constant temperature incubator (Beijing Kewei Yongxing Instrument Co., Ltd., China), Bioprep-24 biological sample homogenizer (Hangzhou Aosheng company, China), H1650 desktop freezing centrifuge (Hunan Xiangyi company, China), KM-250DE desktop ultrasonic cleaner (Kunshan Meimei Ultrasonic Instrument Co., Ltd., China), Millipore pure water instrument (Millipore, USA), ML204 electronic balance (Mettler, Switzerland), circulating vacuum water pump (Gongyi Yuhua Instrument Co., Ltd., China), electrophoresis instrument, electrophoresis tank, and membrane transfer instrument (Beijing 61 Biotechnology Co., Ltd., China).

The following reagents were used in this study: regaloside A (Shanghai Standard Technology Co., Ltd., 0252-dt01), regaloside B (Chengdu PFID Biotechnology Co., Ltd., 18081001), regaloside C (Chengdu PFID Biotechnology Co., Ltd., 18082203), regaloside E (Chengdu PFID Biotechnology Co., Ltd., 18081303), methanol (MS pure; Merck, Germany), and acetonitrile (MS pure; Merck, Germany). Dopamine hydrochloride (batch no.: 100070–201507; 99.8%), noradrenaline tartrate (batch no.: 100169–201404; 94.3%), 5-HT (batch no.: 111656–200401), and glutamic acid (batch no.: 140690–201604; 99.9%) were purchased from CFDA. The CRH, ACTH, cortisol, and IL-1 *β* commercial kits were purchased from Shanghai Crystal Day Biotechnology Co., Ltd. We also used primary antibodies: BDNF (origin: mouse; lot no.: ab205067; Abcam, UK), TrkB (origin: rabbit; lot no.:13129-1-AP; Proteintech, USA), *β*-actin (origin: mouse; lot no.: 66009-1-Ig; Proteintech, USA), secondary antibodies (HRP goat anti-mouse IgG and lot no.: SA00001-1; HRP goat anti-rabbit IgG and lot no.: SA00001-2; Proteintech, USA), RIPA lysis buffer (Shanghai Biyuntian Biotechnology Co., Ltd.), and super ECL plus hypersensitive luminescent solution (Advansta, USA). Fluoxetine capsule was purchased from Lilai Pharmaceutical Co., Ltd. (no. b14201010566); lily herb (Hunan Longshan Heshun Baihe planting base, 20181201) was identified as the dried bulb of *L. lancifolium* Thunb. by Professor Zhou Xiaojiang, Department of Traditional Chinese Medicine Identification, Hunan University of Chinese Medicine; and hen egg yolk was acquired from Hunan Zhongyin ecological breeding professional cooperative (food license no.: jy14307210192437, China).

BHT was prepared as follows: considering a mass ratio of lily to hen egg yolk of 2:1, the appropriate amount of lily herb was diluted in 10 times the amount of distilled water and allowed to extract for 1 h by water decoction. The water decoction was collected, and the extraction was repeated for 1 h by adding 5 times the volume of water. The two extracts of water decoction were combined, concentrated to one volume under reduced pressure, and the hen egg yolk was added at 50°C and mixed, and the mixture was heated for 8 min to prepare BHT and stored at 4°C until use.

### 2.3. Animals

Male Sprague Dawley rats (230–250 g) were provided by Hunan Slake-Jingda Experimental Animal Co., Ltd. (no. 4300470055913; Hunan, China). Rats were housed in a specific pathogen-free experimental animal center of the First Affiliated Hospital of Hunan University of Chinese Medicine (FAH of HNUCM) and maintained under a 12 h light/dark cycle at room temperature (25 ± 1°C) and humidity of 40%–70%. Rats were provided with a standard diet and water ad libitum. All animal experiments were performed according to the protocol approved by the animal care and use committee of FAH of HNUCM.

### 2.4. Experimental Protocol

A total of 72 rats were randomly divided into six groups according to their weight (12 per group): control, chronic unpredictable mild stress (CUMS) model, CUMS + fluoxetine (1.8 mg/kg), CUMS + BHT (2.7 g/kg), CUMS + BHT (5.4 g/kg), and CUMS + BHT (10.8 g/kg) groups. The daily dosage of BHT is recorded in the synopsis of the Golden Chamber in the Eastern Han Dynasty of China. Rats in the control and CUMS groups were intragastrically administered isovolumic distilled water (10 mL/kg·d). After acclimatization for 5 days, rats were exposed to CUMS for 30 consecutive days, and BHT or fluoxetine was simultaneously administered intragastrically once daily in the CUMS + BHT and CUMS + fluoxetine groups, respectively. Rats were subjected to the following stressors for 30 days to trigger CUMS: water deprivation (24 h), food deprivation (24 h), overnight illumination, ice-water bath (4°C, 5 min), tail pinch (1 min, 1 cm from the beginning of the tail), damp sawdust (200 mL of water in 100 g of sawdust bedding), exposure to an empty cage (24 h), cage tilting (45°), inversion of the light/dark cycle, and white noise (3 h). Rats were exposed to these stressors in a random order to ensure the unpredictable characteristics of the experiment.

After the behavioral test was completed, the rats were fasted for 24 h and anesthetized with 3% pentobarbital (30 mg/kg). The rats were fixed on the operating table. Blood samples were collected, left to stand for 1 h, and centrifuged at 3,000 rpm/min for 15 min; the supernatant was collected into 1.5 mL EP tubes. Serum/plasma was collected and stored in a −80°C ultra-low temperature refrigerator until use.

After blood collection, the rats were divided into two groups. Rats in the first group were infused with paraformaldehyde, and then the brain was dissected and placed in a cryotube at 4°C until use. In the second group, the hippocampus was removed, placed directly on ice, and stored at −80°C until analysis.

### 2.5. Behavioral Experiments

#### 2.5.1. Sucrose Preference Test (SPT)

The SPT was conducted before and after CUMS. Rats were provided with two bottles of water, one containing 1% sucrose and one containing distilled water, for 1 h, and the positions of the two bottles were exchanged after 30 min. Sucrose preference was calculated as follows:

(1) $$\begin{array}{c} \text{sucrose preference }=\;\frac{\text{sucrose consumption}}{\left(\text{sucrose consumption }+\text{ distilled water consumption}\right)*100\%}. \end{array}$$

#### 2.5.2. Open-Field Test (OFT)

After 30 days of CUMS, rats of each group were placed in a black open box, with a 70 W incandescent lamp hanging above its center. The length, width, and height of the open box used for testing were 80, 80, and 40 cm, respectively. The bottom of the box was divided into 25 small squares, on average. Each animal was placed in the box, was allowed to adapt for 1 min, and the number of horizontal crossings and vertical erections within 4 min was recorded. The experiment was conducted in a quiet and dark environment, reflecting the autonomous behavior, exploratory behavior, and tension degree of the animals in a new and different environment to evaluate their depressive symptoms.

### 2.6. Hematoxylin/Eosin Staining in the CA1 and Dentate Gyrus (DG) of the Hippocampus

The rat brain tissue in 2.4 was fixed in 4% paraformaldehyde solution overnight, embedded in conventional paraffin, and sectioned into thin slices. Slices were washed with xylene and ethanol baths of successive grades. After washing, the sections were stained with hematoxylin aqueous solution for 5 min, separated with acid water and ammonia water, rinsed with running water and then with distilled water, and stained with eosin alcohol for 2 min. Finally, they were dehydrated with pure alcohol and made transparent by xylene, and the transparent sections were sealed with Canadian gum and observed under a microscope.

### 2.7. Detection of CRH, ACTH, CORT, and IL-1 *β* by ELISA

The plasma of the rats was collected to detect markers associated with the HPA axis. The concentrations of CRH, ACTH, CORT, and Il-1*β* were detected with ELISA, following the manufacturer's instructions. The samples, standard products, and horseradish peroxidase-labeled detection antibodies were added to precoated plates with CRH, ACTH, CORT, and Il-1*β* antibodies and washed thoroughly after incubation. The TMB substrate was used for color development; TMB is converted to blue under the catalysis of peroxidase and finally to yellow upon the addition of sulfuric acid. The color intensity positively correlates with each substance content in the plasma.

### 2.8. Determination of Dopamine (DA), Norepinephrine (NE), Serotonin (5-HT), and Glutamic Acid (Glu) Concentrations in the Hippocampus by UPLC Coupled with Triple Quadrupole (QQQ)/MS

DA, NE, and 5-HT were accurately weighed and dissolved in methanol (0.1% formic acid) to obtain stock solutions of reference. The appropriate amount of Glu was dissolved in ultra-pure water (0.1% formic acid) to obtain a stock solution of reference. Finally, a proper amount of the stock solution was added into the mobile phase of acetonitrile (A) and 0.1% formic acid water (B) (A:B = 2:98) to obtain a series of mixed reference solutions (for DA, NE, 5-HT: 0.001, 0.005, 0.01, 0.05, 0.1, 0.5, 1.0 *μ*g/mL; for Glu: 0.5, 1, 2, 5, 10, 20, and 50 *μ*g/mL).

According to the literature [21], the sample solution was prepared as follows. Hippocampal samples were precisely weighed (50–60 mg), and 1,000 *μ*L of ice methanol (containing 0.1% formic acid, 0°C) plus 10 *μ*L of 3,4-dihydroxybenzylamine (10 *μ*g/mL methanol solution) were added to each sample. Samples were homogenized on ice for 1 min and centrifuged at 4°C for 15 min at 15,000 rpm/min, and the supernatant was collected. The solvent was removed by nitrogen purging, and 100 *μ*L of acetonitrile-0.1% formic acid (98:2 ratio, 0°C) was added, mixed by vortex, and centrifuged at 15,000 rpm/min at 4°C for 15 min, and the supernatant was collected; 10 *μ*L of the supernatant was used for UPLC-QQQ/MS detection.

Chromatography conditions: the chromatographic column was Waters ACQUITY UPLC BEH C18 (2.1 mm × 100 mm, 1.7 m). The mobile phase was acetonitrile (A) and 0.1% formic acid water (B), gradient elution: 0–4 min, 2% A; 4–6 min, 2%∼80% A; 6–8 min, 80%–90% A; 8–10 min, 90% A; column temperature: 30°C; and flow rate: 0.1 mL/min.

MS conditions: the drying temperature was 325°C, the drying gas flow rate was 10 L/min, the capillary voltage was 4.0 kV, the fragment or voltage was 380 V, and the atomization pressure was 35 PSI. Electrospray ionization (ESI) was used to perform MS multiple response monitoring (MRM) scanning in the positive ion detection mode, and MRM was quantified by the retention time and MRM fragments in the control sample.

### 2.9. Western Blot Analysis of BDNF/TRKB Levels in the Hippocampus

RIPA lysis buffer was added to frozen hippocampal tissue; the samples were homogenized by electricity and centrifuged; and the supernatant was collected. A 4.8% stacking gel and a 10% separating gel were prepared, and bromophenol blue was added to the samples before loading into the gel. The electrophoresis was terminated when the bromophenol blue dye reached the bottom of the gel. The protein molecules in the gel were transferred to a nitrocellulose membrane, which was sealed and incubated at room temperature for 150 min, and overnight at 4°C. After washing the membrane with PBST, the corresponding primary antibodies, diluted according to a certain ratio with 1 ^*∗*^ PBST (BDNF: 1 ug/ml, TrkB:1:1000, and *β*-actin: 1:5,000), were added to the membrane and then incubated at room temperature for 90 min. After washing the membrane three times with PBST, the diluted secondary antibodies (HRP goat anti-mouse: 1:5,000 and HRP goat anti-rabbit: 1:6,000) were added and incubated with the membrane at room temperature for 90 min. After washing three times with PBST, the membrane was incubated with ECL chemiluminescence solution for 1 min. The liquid was removed with filter paper, and the hybridized membrane was wrapped in plastic film and exposed to an X-ray film in a dark box. After developing and rinsing, the exposed film was scanned, and the gray value of the target strip was analyzed using the Quantity One professional analysis software.

### 2.10. 16S rRNA Sequencing Analysis of Intestinal Microbial Diversity

The contents of the rectum of the rats of each group were collected aseptically in a cryopreserved tube and immediately stored in liquid nitrogen. After 2 h, the samples were removed and sent to Shanghai Biotree Biotechnology Co., Ltd. with dry ice for 16S rRNA sequencing.

### 2.11. Characteristic Components of BHT

#### 2.11.1. Analysis of Chemical Components in BHT by UPLC-Q-TOF-MS

*(1) Chromatographic Conditions*. The chromatographic column was a Thermo Hypersil Gold C18 column (100 mm × 2.1 mm, 1.9 *μ*m); the following specifications were used: mobile phase: acetonitrile-0.5% acetic acid, gradient elution; flow rate: 0.25 mL/min; column temperature: 30°C; wavelength: 309 nm; and injection volume: 2.00 *μ*L. The conditions for UPLC-MS/MS (negative ion mode) were as follows: ionization mode: electrospray ionization (ESI), ESI-capillary voltage: 2.8 kV, cone voltage: 28 V, and cone gas flow: 50 L/h; ESI + capillary voltage: 3.0 kV, cone voltage: 30 V, and cone gas flow: 50 L/h. The ESI-ionization drying temperature was: *T* = 350°C and *V* = 600 L/h. The flow gradient elution procedure is shown in Table 1.

*(2) Preparation of the Test Solution*. Precisely 5 mL of BHT was absorbed into a 50 mL volumetric flask, and 10 mL of 95% ethanol was added. The mixture was subjected to ultrasound for 30 min and filtered, and the filtrate was filtered again through a 0.22 *μ*m microporous membrane; the subsequent filtrate was collected as the test solution.

*(3) Preparation of the Reference Solution*. The reference substances, regalosides A, B, C, and E, were precisely weighed into a measuring flask, dissolved, and diluted with 80% ethanol to obtain a mixed reference solution containing 46.4 *μ*g/mL regaloside A, 52.8 *μ*g/mL regaloside B, 52.1 *μ*g/mL regaloside C, and 53.2 *μ*g/mL regaloside E.

#### 2.11.2. Content Determination by HPLC-UV

*(1) Preparation of Test and Control Solutions*. The same procedure as in paragraph 2.11.1 was followed.

(2) *Chromatographic Conditions*. The chromatographic column was Agilent Eclipse Plus C18 (2.1 mm × 50 mm, 1.8 *μ*m), with a mobile phase consisting of acetonitrile (A) and 0.1% aqueous phosphate (B), at an A:B ratio of 15:85 (isocratic elution). The flow rate was 1.0 mL/min; the detection wavelength was 309 nm; the injection volume was 10.0 *μ*L; and the column temperature was 30°C.

### 2.12. Statistical Data Analysis

Experimental data are expressed in the form of “*x* ± *s*,” and the statistical software GraphPad Prism 7.0 was used for data analysis. If the data conformed to a normal distribution and homogeneity of variance, one-way analysis of variance and the least significant difference method were used for intergroup comparison, and Tamhane's method was used for data with nonhomogeneity of variance. If the data were not normally distributed, the Kruskal–Wallis H test was used. Significance was set at *α* = 0.05.

## 3. Results

### 3.1. Network Pharmacology to Predict the Efficacy of BHT

After removing compounds lacking target prediction and repeated data, screening was carried out on the SwissADME database, and 28 active compounds of lily and egg yolk with better druggability, as well as 254 targets, were finally selected. The drug-chemical-target network was plotted in Cytoscape software, as shown in Figure 1(a). Through topological analysis of the network of “drug-chemical-target,” we found that the obtained topological parameters were two times the median value of the degree, which was 10, and the median values of BC and CC were 0.003235205 and 0.3559322, respectively. Molecules satisfying the above values for these three parameters were determined as the key pharmacodynamic molecules. The higher the degree value of the compound, the more potential targets it has. Key pharmacodynamic molecules with high values such as *β*-sitosterol (mol4), oleic acid (mol5), stigmasterol (mol19), regaloside E (mol27), regaloside A (mol25), regaloside B (mol26), Glu (mol16), and arachidonic acid (mol9) may play an important role in the course of drug treatment.  Table 2 shows the key pharmacodynamic molecules and their topological parameters in the network of “drug-compound-target”.

A total of 75 predicted targets were obtained from the intersection of drug targets of lily and hen egg yolk with depressive disease targets; these were imported into the STRING database, and network data with confidence >0.7 were imported into Cytoscape software to generate an interaction network plot of potential targets for lily and hen egg yolk for the treatment of depression, as shown in Figure 1(b). Then, topological analysis was performed. The double value of the median degree was 14; the median BC was 0.01058337; and the median CC was 0.42307692. Twelve targets that met the above parameters were identified as the core targets (Table 3). Figure 1(b) and Table 3 show that mitogen-activated protein kinase 1 (MAPK1), AKT1, BDNF, tumor necrosis factor, vascular endothelial growth factor A, and other targets had high values for these topological parameters and could thus play a central role in the antidepression effects of lily and egg yolk.

Enrichment analysis was carried out using DAVID and *P* < 0.05. The results showed 322 GO entries, 241 biological process entries, 35 cell component entries, and 46 molecular function entries. The top 20 GO terms with the lowest *P* values were selected to plot in a chart (Figure 2(a)). Among them, response to estradiol, steroid hormone receptor activity, response to glucocorticoids, positive regulation of nitric oxide biosynthetic process, positive regulation of MAPK activity, positive regulation of peptidyl-serine phosphorylation, ionotropic glutamate receptor signaling pathway, ionotropic glutamate receptor activity, negative regulation of synaptic transmission, glutamatergic, and neuronal cell body showed high enrichment degrees.

Reactome pathway analysis revealed a total of 190 pathways related to depression (Figure 2(b)). The top-ranked pathways were signaling by receptor tyrosine kinases, glutamate binding, activation of AMPA receptors, synaptic plasticity, negative regulation of the PI3K/AKT network, BDNF-activated NTRK2 (TRKB) signaling, mTOR signaling, and so on.

The network “drug-compound-target pathway” was constructed in Cytoscape software, as shown in Figure 3. The blue node represents the predicted target; the yellow represents the core target; the red represents the pathway; and the green and orange nodes represent the drug and its pharmacophore, respectively. Different targets of 28 active components of lily and hen egg yolk acted on different pathways, indicating that the mechanism of action of lily and hen egg yolk in the treatment of depression involves multiple components, targets, and pathways. As shown in Figure 3, the target pathway of BHT mainly involved BDNF/TrkB and PI3K/AKT signaling pathways, glutamate binding, synaptic transmission based on neurotransmitter signal, and response to glucocorticoids.

### 3.2. Behavioral Results

#### 3.2.1. Sucrose Preference Test

**Figure 4:**
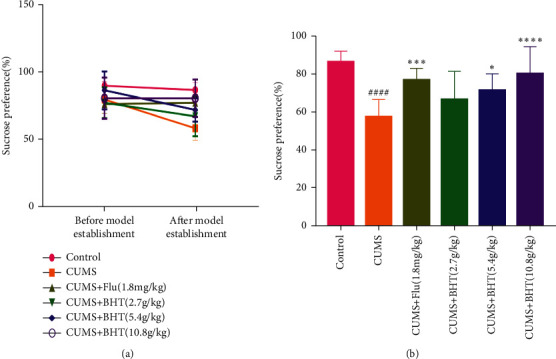
SPT of rats. (a) SPT comparison before and after CUMS and (b) SPT comparison of each group after CUMS. Control represents the normal control group; CUMS represents the CUMS group; CUMS + Flu represents the intervention of 1.8 mg/kg fluoxetine on CUMS rats; and CUMS + BHT represents the intervention of 2.7 g/kg, 5.4 g/kg, and 10.8 g/kg BHT on CUMS rats. The SPT value of CUMS rats increased significantly after the intervention. The statistical method was one-way ANOVA (*n* = 12). Compared with the control group, ^####^*P* < 0.001, and compared with CUMS, ^*∗*^*P* < 0.05,^*∗∗*^*P* < 0.005,^*∗∗∗*^*P* < 0.001.

The SPT is an important index used to evaluate anhedonia in depressed animals. The sucrose consumption of rats was tested before and after depression model generation, and the results are shown in Figure 4.  Figure 4(a) compares the change trend of sucrose consumption in rats in the same group before and after CUMS; the sucrose preference rate of rats in each group was minimally different before CUMS, while after CUMS, the rates of the control, fluoxetine, and CUMS + 10.8 g/kg BHT groups were similar to those before (Figure 4(a)). After 30 days of chronic stress, the sucrose preference rate in rats in the CUMS group was significantly lower than that in the control group (*P* < 0.001; Figure 4(b)). The rate in the 10.8 g/kg BHT group was significantly higher than that in the CUMS group (*P* < 0.001), while the rates in the fluoxetine and 5.4 g/kg BHT groups were also significantly higher (*P* < 0.005, *P* < 0.05); there was no statistical difference for the 2.7 g/kg BHT group.

#### 3.2.2. Open-Field Test Results

The OFT is a method to evaluate autonomous and exploratory behavior and stress in experimental animals based on the frequency and duration of some behaviors in the novel environment.

**Figure 5:**
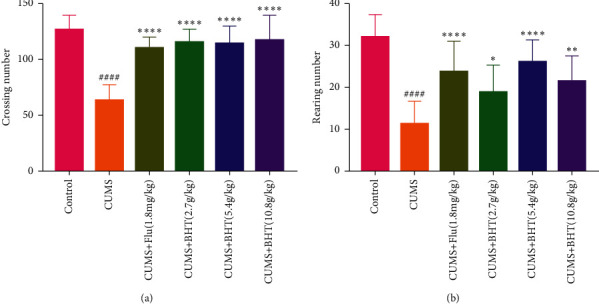
Open-field test of rats. (a) The number of horizontal crossing of rats in each group and (b) the number of vertical standing of rats. In the plot, Control represents the normal control group; CUMS represents the CUMS group; CUMS + Flu represents the intervention of 1.8 mg/kg fluoxetine on CUMS rats; and CUMS + BHT represents the intervention of 2.7 g/kg, 5.4 g/kg, and 10.8 g/kg BHT on CUMS rats. The statistical method was one-way ANOVA (*n* = 12). Compared with the control group, ^####^*P* < 0.001, and compared with CUMS, ^*∗*^*P* < 0.05,^*∗∗*^*P* < 0.01,^*∗∗∗*^*P* < 0.001.

As shown in Figure 5(a), the number of horizontal crossings was significantly lower in the CUMS than in the control group (*P* < 0.001), whereas that in the all-drug groups increased significantly (*P* < 0.001). After 30 days of chronic stress, the number of vertical erections was significantly lower among rats in the CUMS group than those in the control group (*P* < 0.001; Figure 5(b)). Horizontal crossing times were significantly greater among rats in the fluoxetine, CUMS + 2.7 g/kg BHT, CUMS + 5.4 g/kg BHT, and CUMS + 10.8 g/kg BHT groups than in those in the CUMS group (*P* < 0.001, <0.05, <0.001,  and < 0.01, respectively.

Collectively, the above results indicate that the TCM diet formula BHT can improve the depressive symptoms of rats exposed to CUMS, especially at doses of 5.4 g/kg.

### 3.3. Effects of BHT on Pathologic Structural Deficits in the Hippocampus of Depressed Rats

#### 3.3.1. Effects on the Hippocampal CA1 Region of Depressed Rats

**Figure 6:**
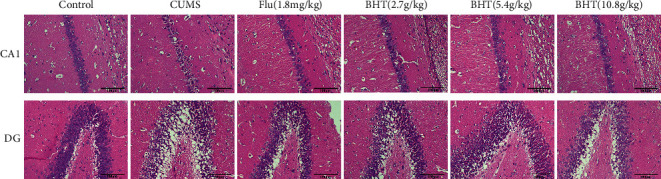
Pathological changes in hippocampus CA1 and DG areas of rats by HE staining. Control represents the normal control group; CUMS represents the CUMS group; Flu represents the intervention of 1.8 mg/kg fluoxetine on CUMS rats; and BHT represents the intervention of 2.7 g/kg, 5.4 g/kg, and 10.8 g/kg BHT on CUMS rats (*n* = 6). Note: red arrow indicates pyknosis cell and green arrow indicates normal neurons (scale bar = 100 *μ*m).

Based on HE staining, neurons in the CA1 area of the hippocampus of the control group had complete structure, regular cell morphology, normal intercellular space, and orderly and dense arrangement; 2–3 layers of cells were visible; and nucleoli were apparent. Compared with the CA1 neurons in the control group, those in the CUMS group were atrophic and arranged loosely; most of the cells were vacuoles; and the nuclei were rigid. Compared with the CUMS group, the neuronal structure, cell morphology, and intercellular space in the hippocampal CA1 area obviously improved in the fluoxetine and CUMS + 2.7/5.4/10.8 g/kg BHT groups; among BHT groups, the CUMS + 5.4 g/kg BHT group showed the most obvious improvements (Figure 6).

#### 3.3.2. Effects on the Hippocampal DG in Depressed Rats

DG neurons in the hippocampus of the control group were arranged regularly; the cells were granular; and the chromatin was evenly distributed. In contrast, the DG neurons of rats in the CUMS group were lost and sparsely arranged, and chromatin was unevenly distributed. The DG neurons in the hippocampus of the drug groups were arranged more regularly, and the chromatin was more evenly distributed. In general, the improvement effect in neurons in the DG area of the hippocampus was more obvious in the fluoxetine and CUMS + 5.4 g/kg BHT groups. The results are shown in Figure 6.

### 3.4. Neuroendocrine HPA Axis Indicators

**Figure 7:**
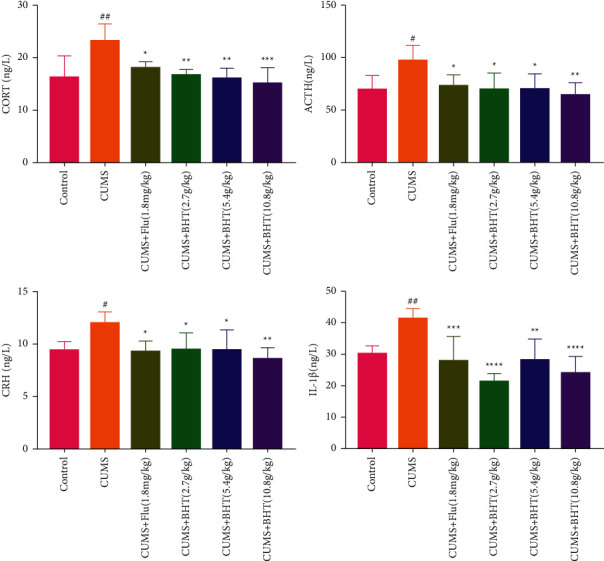
Levels of CORT, ACTH, CRH, and IL-1*β* in rats plasma (*x* ± *s*; *n* = 6, ng/L). Compared with the control group, ^#^*P* < 0.05,^##^*P* < 0.01, and compared with CUMS, ^*∗*^*P* < 0.05,^*∗∗*^*P* < 0.01,^*∗∗∗*^*P* < 0.005,^*∗∗∗∗*^*P* < 0.001.

As shown in Figure 7, the plasma levels of CRH, ACTH, CORT, and Il-1*β* were significantly higher in the CUMS group than in the control group (*P* < 0.01 or *P* < 0.05). However, the corresponding levels in the CUMS + BHT groups were significantly lower than those in the control group (*P* < 0.05, *P* < 0.01, or *P* < 0.005).

### 3.5. Neurotransmitter Targeted Metabolomics

#### 3.5.1. The Standard Curve

UPLC-QQQ/MS analysis was performed on serial concentrations of the mixed reference substances, as described in paragraph 2.8, and the chromatographic peak area was recorded. With the peak area as the ordinate and the reference concentration as the abscissa, the standard curve was drawn, and linear regression was performed. The linear regression equations for DA, NE, 5-HT, and Glu were obtained as follows: DA, *Y* = 3008.5*x*−70.842 (*r* = 0.9954) and linear range = 0.001–1.0 g/mL; NE, *Y* = 10.484*x*−0.9794 (*r* = 0.9981) and linear range = 0.001–1.0 g/mL; 5-HT, *Y* = 74.796*x*−0.7164 (*r* = 0.9952) and linear range = 0.001–1.0 g/mL; and Glu, *Y* = 468.93*x*−69.387 (*r* = 0.9999) and linear range = 0.5–50.0 g/mL. The linear regression equation and linear range are shown in Table 4.

#### 3.5.2. Analysis of Neurotransmitter Content

**Figure 8:**
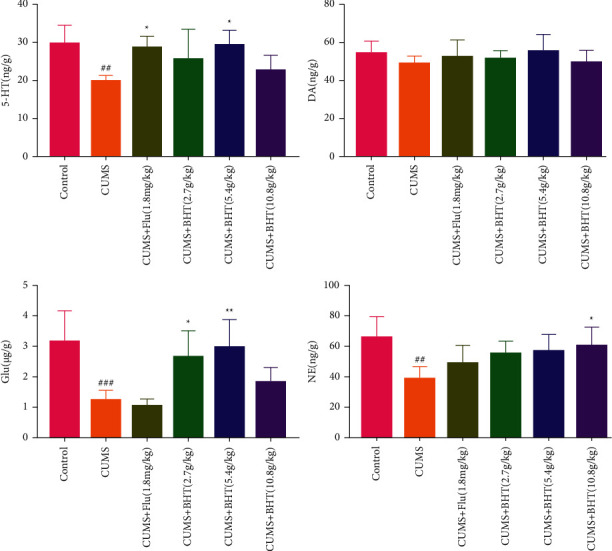
Differences of four neurotransmitters in the hippocampus of rats. Control represents the normal control group; CUMS represents the CUMS group; CUMS + Flu represents the intervention of 1.8 mg/kg fluoxetine on CUMS rats; and CUMS + BHT represents the intervention of 2.7 g/kg, 5.4 g/kg, and 10.8 g/kg BHT on CUMS rats. The statistical method was one-way ANOVA (*n* = 6). Compared with the control group, ^###^*P* < 0.005,^##^*P* < 0.01, and compared with the CUMS, ^*∗*^*P* < 0.05,^*∗∗*^*P* < 0.01.

The results regarding the concentration of DA, NE, 5-HT, and Glu in hippocampal samples of rats in each group are shown in Figure 8. The concentration of NE, 5-HT, and Glu in the hippocampus was significantly lower in the CUMS than in the control group (*P* < 0.01). The levels of 5-HT were significantly higher in the fluoxetine and CUMS + 5.4 g/kg BHT groups (*P* < 0.05); those of Glu were significantly higher in the CUMS + 2.7 g/kg and CUMS + 5.4 g/kg BHT groups (*P* < 0.05); those of NE were significantly higher in the CUMS + 10.8 g/kg BHT group than in the CUMS group (*P* < 0.05); and the effect of 5.4 g/kg BHT on the level of NE was close to that of 10.8 g/kg BHT; there was no statistical difference in the content of DA in the hippocampus among groups. Different doses of BHT had different effects on different neurotransmitters, and the effect of 5.4 g/kg BHT was better in general.

### 3.6. BDNF and TrkB Levels in Hippocampal Tissue Samples

**Figure 9:**
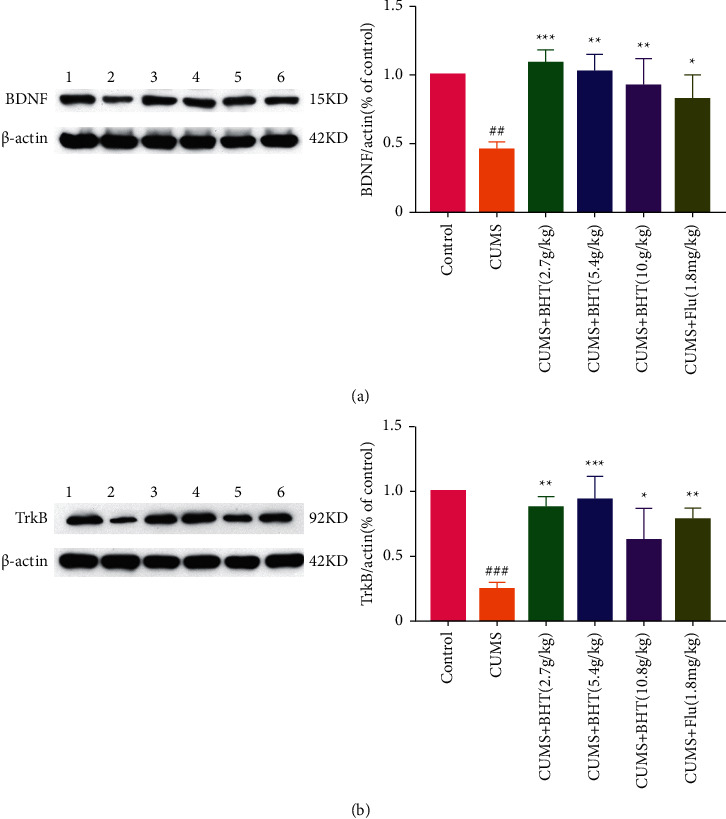
Expression level of BDNF and TrkB in the hippocampus of rats. No. 1–6 in protein band plot correspond to the Control group, CUMS group, CUMS + BHT 2.7 g/kg group, CUMS + BHT 5.4 g/kg group, CUMS + BHT 10.8 g/kg group, and CUMS + Flu 1.8 mg/kg group, respectively. Control represents the control group; CUMS represents the CUMS group; CUMS + Flu represents the intervention of 1.8 mg/kg fluoxetine on CUMS rats; and CUMS + BHT represents the intervention of 2.7 g/kg, 5.4 g/kg, and 10.8 g/kg BHT on CUMS rats. (a) Quantitative analysis of BDNF protein expression in hippocampus after treatment of BHT. (b) Quantitative analysis of TrkB protein expression in hippocampus after treatment of BHT. The statistical method was one-way ANOVA (*n* = 3). Compared with the control group, ^##^*P* < 0.01,^###^*P* < 0.005, and compared with the CUMS, ^*∗*^*P* < 0.05,^*∗∗*^*P* < 0.01,^*∗∗∗*^*P* < 0.005.

After continuous intervention with BHT for 30 days, the expression levels of BDNF and TrkB in the hippocampal tissues of each group were analyzed (shown in Figure 9). Compared with the control group, the protein expression levels of BDNF and TrkB were significantly reduced in the CUMS group (*P* < 0.01, *P* < 0.005). Compared with the CUMS group, the expression levels of BDNF were significantly increased in the CUMS + 2.7 g/kg BHT group (*P* < 0.005) and CUMS + 5.4 g/kg and 10.8 g/kg BHT groups (*P* < 0.01) and also increased in the CUMS + fluoxetine group, and the difference was statistically significant (*P* < 0.05). Compared with the CUMS group, the protein expression levels of TrkB were increased most significantly in the CUMS + 5.4 g/kg BHT group (*P* < 0.005) and then in the CUMS + 2.7 g/kg BHT and CUMS + fluoxetine groups (*P* < 0.01). CUMS + 10.8 g/kg BHT could also increase the expression of TrkB with a statistically significant difference (*P* < 0.05).

### 3.7. 16S rRNA Sequencing Results of Intestinal Microorganisms

**Figure 10:**
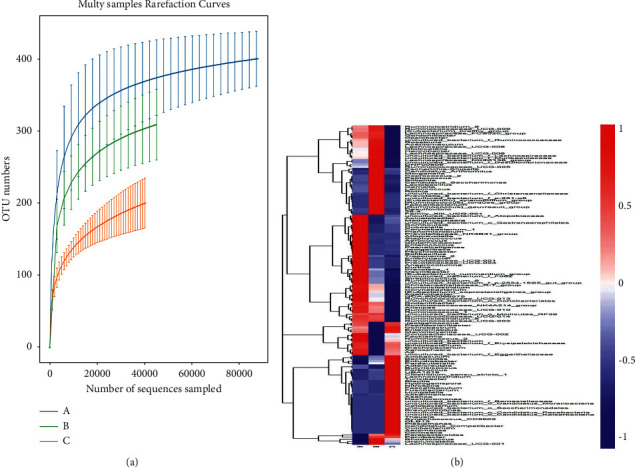
(a) Dilution curve of samples: the curve rises flatly, indicating that the species in this environment do not increase significantly with the increase in the number of sequences sampled. So the sample sequence was sufficient, and data analysis could be carried out. (b) Heat map of sample abundance: the abscissa indicates grouping, the ordinate represents genus, red indicates high abundance, and blue indicates low abundance. Color changes and similarities in the heat map directly represent differences in community composition at the genus level among the control, CUMS, and BHT groups (CUMS + 5.4 g/kg).

Using UCLUST in QIIME [22, 23] (version 1.8.0) software, tags were clustered at a 97% similarity level to obtain operational taxonomic units (OTUs), annotated based on Silva (bacteria) and UNITE (fungi) taxonomic databases, resulting in 493, 472, and 436 OTUs for the control, CUMS, and CUMS + 5.4 g/kg BHT groups, respectively. Based on the results of OTU analysis,14 phyla, 22 classes, 36 orders, 65 families, 143 genera, and 155 species were obtained. The dilution curve allows representing the number of species by randomly selected sequences in the sample. The curve was constructed with the number of sequences and species to verify whether the amount of sequencing data was sufficient to reflect the species diversity in the sample, thus indirectly reflecting the species richness in the sample. Within a certain range, if the curve rises sharply, increases in the number of sequences sampled indicate a large number of species in the community; in contrast, a flat curve indicates that the species in this environment do not increase significantly with the increase in the number of sequences sampled. As shown in Figure 10(a), the sample sequence was sufficient, and data analysis could be conducted.

**Figure 11:**
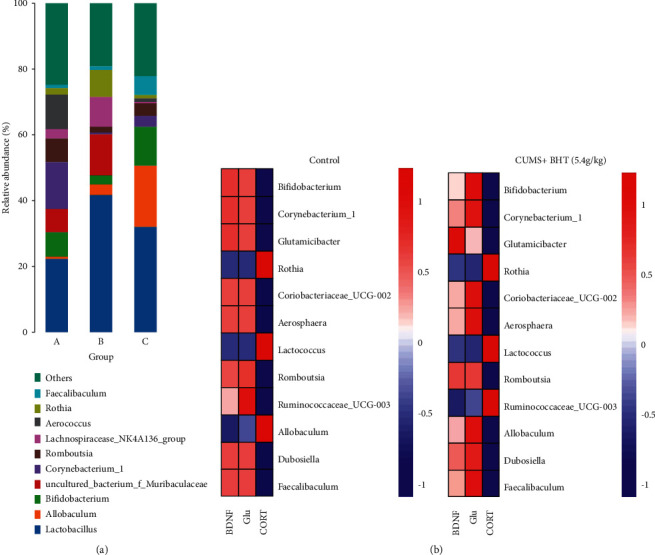
Similarities and differences of community composition of different species at the genus level. (a) Correlation analysis between BDNF, Glu, and CORT with 12 Enterobacteriaceae with the lowest *P* value and (b) left plot: CUMS + BHT (5.4 g/kg) group and right plot: control group. The abundances of *Lactobacillus* and *Bifidobacterium*, which are the dominant flora, in the control, CUMS, and BHT groups were high. Note: the ordinate shows 16S flora; the abscissa shows the detection index; red (corr = 1) indicates positive correlation; blue (corr = −1) indicates a negative correlation; and white indicates irrelevant (corr = 0); the darker the color, the stronger the correlation.

Figure 10(b) shows the heat map of relative abundance of Enterobacteriaceae detected at the genus level, as generated by *Z*-score transformation. In the map, the abscissa indicates grouping; the ordinate represents genus; red indicates high abundance; and blue indicates low abundance. Color changes and similarities in the heat map directly represent differences in community composition at the genus level among the control, CUMS, and BHT groups (CUMS + 5.4 g/kg BHT). In area A, the color of the heat map of the control and BHT groups was blue, while that of the CUMS group was red; in area B, the color of the heat map of the control and BHT groups was red, while that of the CUMS group was blue; thus, the relative abundance of genus-level enterobacteria in these two areas was quite different. The relative abundances of the top 10 genus-level enterobacteria were further analyzed and are plotted in Figure 11(a). The abundances of *Lactobacillus* and *Bifidobacterium*, which are the dominant flora, in the control, CUMS, and BHT groups were high; the abundance of *Lactobacillus* in the CUMS group was as high as 41.7%, significantly higher than those in the control and BHT groups; and the abundance of *Bifidobacterium* in the BHT group was 11.5%, 7.5% in the control group, and only 2.8% in the CUMS group (*P* < 0.01). The abundance of *Allobaculum* in the BHT group was 18.7%, while that in the CUMS and control groups was 3.2% and 0.6%, respectively (*P* < 0.01, *P* < 0.05). The abundance of uncultured bacterium *F. Muribaculaceae* in the control and BHT groups was significantly lower than that in the CUMS group (*P* < 0.05). These results suggest that BHT may improve intestinal flora disorder in depressed rats.

A correlation coefficient is a strength that indicates a statistical relationship between different variables. Its values range between −1 and 1, that is, strong negative correlation (−1), completely uncorrelated (0), and strong positive correlation (1). If the relationships among the variables in the data are to be analyzed, we can perform a correlation analysis of the variables with the help of the corrplot package in the *R* language. Corrplot is a method for batch analysis, and we can obtain a correlation heat map in which the dark and light colors represent the magnitude of the correlation coefficient. In this research, we selected the content of CORT, Glu, BDNF, and the relative abundance of 12 Enterobacteriaceae with the lowest *P*-value to generate heat maps for correlation analysis. In Figure 11(b), the ordinate shows 16S flora; the abscissa shows the detection index; red (corr = 1) indicates a positive correlation; blue (corr = −1) indicates a negative correlation; and white indicates irrelevant (corr = 0); the darker the color, the stronger the correlation. CORT negatively correlated with nine Enterobacteriaceae (*Bifidobacterium*, *Glutamicibacter*, etc.), and Glu and BDNF positively correlated with it, while *Rothia* and *Lactobacillus* showed an opposite trend.

### 3.8. Chemical Constituents in BHT

#### 3.8.1. Analysis of Chemical Components in BHT by UPLC-Q-TOF-MS

**Figure 12:**
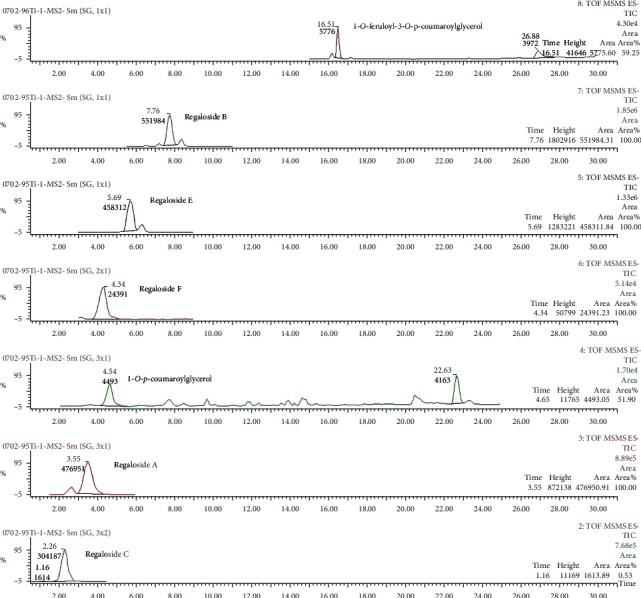
Daughter ion flow graph of the MS-MS spectrogram of BHT in negative ion modes. *t* = 2.26 min, regaloside C; *t* = 3.55 min, regaloside A; *t* = 4.34 min, regaloside F; *t* = 4.65 min, 1-o-p-coumaroylglycerol; *t* = 5.69 min, regaloside E; *t* = 7.76 min, regaloside B; and *t* = 16.51 min, 1-o-feruloyl-3-o-p-coumaroylglycerol.

Ultrasonic extracts of samples were analyzed by UPLC-Q-TOF-MS. At 309 nm, many effective components in BHT showed large absorption peaks; thus, this wavelength was used for qualitative analysis and detection. The main chemical components of BHT were better separated by secondary MS. Through the analysis of primary and secondary MS, combined with literature and reference identification, the structure was identified. In the negative ion mode, seven chemical components were identified as regalosides A, B, C, E, and F; 1-*o*-*p*-coumaroylglycerol; and 1-*o*- feruloyl-3-*o*-*p*-coumaroylglycerol.  Figure 12 and Table 5 show the specific mass charge ratio and fragment ion information .

#### 3.8.2. Content Determination by HPLC-UV

*(1). Methodology Validation*

**Figure 13:**
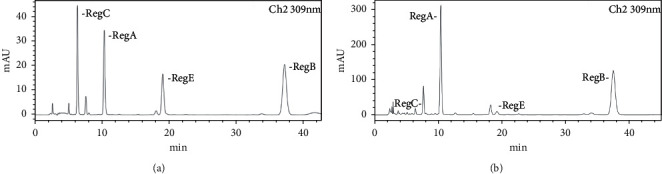
HPLC of regalosides A, B, C, and E in BHT samples. (a) Reference solution (309 nm) and (b) test solution (309 nm).

1. Standard curve experiment: HPLC analysis was performed according to the conditions described in paragraph 2.11.2; the results are shown in Figure 13. Based on the *R* values, good linear relationships within their respective linear ranges were obtained for all regalosides. The corresponding linear regression equations and linear ranges were as follows: regaloside A, *y* = 10934*x*−15291 (*r* = 0.9978, 4.0–46.4 *μ*g/mL); regaloside B, *y* = 15419*x*−31618 (*r* = 0.9958, 5.0–52.8 *μ*g/mL); regaloside C, *y* = 8635.7*x*−2806.8 (*r* = 0.9991, 5.0–52.1 *μ*g/mL); and regaloside E, *y* = 7378.4*x*−237.13 (*r* = 0.9953, 5.0–53.2 *μ*g/mL).
2. Precision test: according to the chromatographic conditions described in paragraph 2.11.2, 10 *μ*L of mixed reference solution was sampled continuously 6 times, and the peak areas obtained were analyzed. The RSD of peak areas of regalosides A, B, C, and E was 1.78%, 0.71%, 0.69%, and 1.86%, respectively, indicating that the instrument's precision was good.
3. Repeatability test: after precise measurement of BHT samples and parallel preparation of six test solutions for HPLC analysis, the results for the RSD of the peak areas were 0.77%, 0.63%, 0.57%, and 0.49% for regalosides A, B, C, and E, respectively, indicating that the method had good repeatability.
4. Stability test: the test solution of BHT samples was prepared according to the method described in paragraph 2.11.2. The solution was placed at room temperature for 0, 2, 4, 8, 12, and 24 h. The samples were analyzed by HPLC as described in paragraph 2.11.2. The results showed that the RSD of the peak areas of regalosides A, B, C, and E in the test solution within 24 h was 0.37%, 0.52%, 0.74%, and 0.67%, respectively, indicating that the method was stable.
5. Sample addition recovery: six parts of the BHT test solution were precisely measured; the appropriate amount of mixed reference solution was added; and the samples were pretreated according to descriptions in paragraph 2.11.2. HPLC-UV recovery test was carried out accordingly. The chromatographic peak area was recorded, and the recovery rate of sample addition was calculated. The results showed that the recovery rates of regalosides A, B, C, and E in BHT were 101.43%, 105.47%, 102.43%, and 100.32%, respectively, while the corresponding RSDs were 1.51%, 1.45%, 2.48%, and 2.09%, respectively, indicating good recovery rates.

*(2) Determination of Four Characteristic Components in BHT by HPLC-UV*. Three samples of six batches of lily medicinal materials were taken, and the test solution was prepared as described in paragraph 2.11.2. The chromatographic peak areas were recorded, and the contents of regalosides A, B, C, and E in the samples were calculated (Table 6 and Figure 13). The results showed that the contents of the main chemical constituents of the BHT decoction were obviously different. In the test samples, the content of regalosides A, B, C, and E was 11.05–13.76 mg/mL, 4.68–9.41 mg/mL, 0.34–0.69 mg/mL, and 0.60–0.87 mg/mL, respectively. The content of regalosides A and B was significantly higher than that of regalosides C and E.

## 4. Discussion

To explore the potential mechanism of BHT on depression, the potential active ingredients, and depression-related targets of BHT were screened by TCMSP, SwissADME, GeneCards, OMIM, and network pharmacology methods, and the related “drug-compound-target” network was constructed using Cytoscape 3.7.2. Meanwhile, with the help of the DAVID, GO biological enrichment analysis was carried out on the predicted targets to construct the network diagram of the “drug-compound-target pathway.” Network pharmacology studies suggested that the BDNF/TrkB signaling pathway, PI3K/AKT signaling pathway, synaptic neurotransmission, and choline metabolism are potential signaling pathways mediating the antidepressant efficacy of BHT. This study shows the characteristics of multicomponent, multitarget, and multipathway analyses, which can provide a reference for experimental animal research on the antidepressive mechanisms of BHT.

To verify the mechanism of BHT effects indicated by network pharmacology prediction, we analyzed differences in depressive behavior, neuroendocrine index of the HPA axis, neurotransmitter levels, hippocampus structural characteristics, intestinal bacteria, and protein BDNF and TrkB expression levels in the hippocampus among rats in different groups. The results showed that CUMS-induced depressive-like behavior in rats hyperactivated the HPA axis, decreased the contents of the neurotransmitters 5-HT, Glu, and NE, induced pathological changes in hippocampal tissue structure, decreased abundance of *Bifidobacterium* at the genus level, and significantly decreased BDNF and TrkB protein expression levels in hippocampal tissue. In contrast, intervention with BHT ameliorated or reversed these effects.

Normal rats are naturally inquisitive, gregarious, and have normal appetites; however, depressed rats have decreased appetite and reduced desire to explore [26]. Additionally, they prefer to be alone in a small space, and their learning and memory abilities decline [27, 28]. The behavior of CUMS rats highly corresponds to the behavior of depressed patients; thus, CUMS is a common method to induce and study depression [29]. In this study, the behavioral evaluation of anhedonia and free social exploration in experimental rats was conducted by SPT and OFT. In our experiment, the sucrose preference of rats in the CUMS group decreased significantly, indicating significant anhedonia. In the OFT, horizontal crossing and vertical erection times significantly decreased in the CUMS group compared to those in the control group. According to the above comprehensive judgment of depression ethology, the CUMS-induced depression model was successfully replicated. After the intervention with BHT, the SPT and OFT results indicated good antidepressant effects of BHT in CUMS rats. Overall, BHT reversed the depression-like behavior.

Hyperfunction of the HPA axis, a neuroendocrine stress response system, positively correlates with the content of CRH, ACTH, and CORT [30]. Overactivation of the HPA axis can lead to neuroinflammation and deficits in the hippocampal structure and function, thus inducing the occurrence of depression or aggravation of depressive symptoms [31]. In this study, we found that the plasma levels of CRH, ACTH, CORT, and Il-1*β* in the BHT group were significantly lower than those in the CUMS group, indicating that BHT has a regulatory effect on the HPA axis.

Neurotransmitters such as 5-HT, NE, DA, and Glu play an important role in the development of depression [32], which can activate the stress loop from bottom to top through the vagus nerve pathway and circulatory system and directly affect the function of the central nervous system. In this study, the content of NE, 5-HT, and Glu in the hippocampus of the CUMS group was significantly lower than in the hippocampus of the control group. 5-HT, Glu, and NE levels were significantly increased by fluoxetine and BHT compared with the respective levels in the CUMS group. The low content of neurotransmitters is recognized by many scholars as the pathogenesis of depression; our experiment also confirmed that the contents of neurotransmitters in the CUMS rats were significantly altered under the intervention of BHT, indicating that BHT is worthy of further study regarding the search for effective treatments against depression.

The hippocampus is an important part of the limbic system of the brain and plays an important role in emotion regulation. Histomorphological changes in the hippocampus can be used as a criterion to judge the degree of depression-induced damages [33, 34]. The hippocampus is divided into the granulosa cell layer and pyramidal cell layer, namely, the dentate gyrus (DG) area and the CA area (ca1-4), respectively. The CA1 area belongs to the stress-prone area, while the DG area belongs to the main neurogenesis area of the hippocampus [35]. Therefore, HE staining was used to study the histopathological structure of CA1 and DG regions to investigate the effects of BHT on depression. The results showed that neurons in the CA1 area of the hippocampus of rats in the CUMS group were atrophic and loosely arranged, and some cells were vacuoles, with their nuclei fixed and contracted; neurons in the DG area were lost and sparsely arranged, and chromatin was unevenly distributed. After the intervention with BHT, the degree of pathological damage in the CA and DG areas was effectively improved.

The intestinal flora not only can affect the HPA axis function through the brain-gut axis, thus affecting the release of cortisol, ACTH, and CRH [36], but can also directly synthesize neurotransmitters, thus regulating the function of intestinal mucosal epithelial cells. For example, *Lactobacillus* and *Bifidobacterium* can produce GABA; *Escherichia*, *Bacillus*, and *Saccharomyces* can produce NE; *Candida*, *Streptococcus*, *Escherichia*, and *Enterococcus* can stimulate the production of 5-HT; and *Corynebacterium glutamate* is the main strain to synthesize Glu [9]. The results of our animal experiments showed that the relative abundance of *Lactobacillus* and *Bifidobacterium*, constituents of the dominant flora, were all high in the control, CUMS, and BHT groups, and however, the abundance of *Lactobacillus* in the CUMS group was significantly higher than that in the control and BHT groups; the abundance of *Bifidobacterium* was significantly higher in the control and BHT groups than in the CUMS group; and the abundance of the uncultured bacterium *F. Muribaculaceae* was significantly lower in the control and BHT groups than in the CUMS group, while the abundance of *Allobaculum* was higher in the BHT group than in the CUMS and control groups. Other studies have shown that *Bifidobacterium* and *Glutamate* bacilli may be positively correlated with the depressive phenotype, and their relative abundance in the feces of depressed patients is reduced, suggesting that it can be used as a general characteristic of depression [37]. In this study, the relative amount of *Bifidobacterium/Glutamicibacter* in the CUMS group was also lower than that in the BHT and control groups. In addition, the Corrplot correlation analysis of different flora with BDNF, Glu, and CORT levels suggested that BHT may improve depressive symptoms by affecting BDNF protein expression, Glu synthesis, and HPA axis activity through enterobacterial co-metabolism by regulation of the brain-gut axis.

## 5. Conclusions

In summary, our network pharmacology studies have predicted that BHT may exert its antidepressant effects by regulating the expression of BDNF/TrkB, synaptic neurotransmission, and neuroinflammation. Further animal experiments confirmed that BHT regulates the expression of BDNF/TrkB, affects the metabolism of neurotransmitters and the diversity of intestinal microbial flora, reduces the hyperactivity of the HPA axis, restores the hippocampal structural deficits, improves depressive behavior, and may play a role in antidepression in a coordinated way. Interestingly, all the raw materials in BHT derive from Chinese traditional medicine and can be used both as food and medicine, which is very suitable for long-term clinical use because depression is a chronic disease of mood disorder, and it needs long-term medication. Moreover, it is the first time that the antidepressant mechanism and pharmacodynamic material basis of BHT are studied. This has important innovation value in explaining the scientific connotation of this TCM prescription.

## Figures and Tables

**Table 1:** Gradient elution procedure.

Time (min)	Mobile phase A, acetonitrile (%)	Mobile phase B, acetic acid (%)
0	12	88
2	12	88
15	30	70
18	90	10
19	90	10
20	12	88

**Table 2:** Key pharmacodynamic molecules and topological parameters in BHT.

Herb	Molecule ID	Molecule name	Molecular weight (g·mol^−1^)	OB (%)	DL	Degree	BC	CC
JZH, BH	MOL4	Beta-sitosterol	414.79	36.91	0.75	21	0.06750618	0.42857143
JZH	MOL5	Oleic acid	282.52	33.13	0.14	19	0.04278457	0.39923954
BH	MOL19	Stigmasterol	412.77	43.83	0.76	17	0.0374903	0.40540541
BH	MOL27	Regaloside E	458.46	7	0.55	15	0.01716852	0.3633218
JZH	MOL9	Arachidonic acid	304.52	45.57	0.2	15	0.03220645	0.39622642
BH	MOL25	Regaloside A	400.42	11.38	0.46	11	0.00810584	0.35353535
BH	MOL26	Regaloside B	442.46	15.63	0.51	11	0.00775733	0.35353535
JZH	MOL16	Glutamic acid	147.15	6.66	0.02	10	0.01255659	0.37366548
BH	MOL28	Regaloside C	416.4	0	0	9	0.00618232	0.34653465
JZH	MOL15	Aspartic acid	133.12	79.74	0.02	7	0.00657208	0.36082474
JZH	MOL1	Palmitic acid	256.48	19.3	0.1	6	0.00520702	0.3633218
JZH	MOL14	Serine	105.11	98.47	0.01	6	0.00475422	0.36082474
JZH	MOL2	Linoleic acid	280.5	41.9	0.14	6	0.00386105	0.3633218
BH	MOL22	3-Demethylcolchicine	385.45	39.34	0.57	6	0.00931012	0.36842105
JZH	MOL17	Leucine	131.2	72.92	0.01	4	0.00166033	0.3559322
JZH	MOL8	Myristic acid	228.42	21.18	0.07	4	0.00196339	0.35836177
JZH	MOL10	Zoomaric acid	254.46	35.78	0.1	3	7.23*E* − 04	0.3559322
JZH	MOL12	Linolenic acid	278.48	45.01	0.15	3	7.23*E* − 04	0.3559322
JZH	MOL3	Ergosterol	396.72	14.29	0.72	3	0.00260936	0.35117057
JZH	MOL6	Stearic acid	284.54	17.83	0.14	3	7.23*E* − 04	0.3559322
JZH	MOL11	Lanosterol	426.8	42.12	0.75	2	0.00105441	0.35117057
BH	MOL20	Isopimaric acid	302.5	36.2	0.28	2	0.00123783	0.35117057
BH	MOL23	26-O-Β-D-Glucopyranosyl-3Β, 26-dihydroxy-5-cholesten-16, 22-dioxo-3-O-Α-L-rhamnopyranosyl-(1⟶2)-Β-D-glucopyranoside_Qt	430.69	35.11	0.81	2	0.00107903	0.33546326
JZH	MOL7	Cholesterol	386.73	37.87	0.68	2	0.00105441	0.35117057
JZH	MOL13	Desmosterol	384.71	13.23	0.68	1	0	0.30259366
JZH	MOL18	Lysine	146.22	29.33	0.02	1	0	0.30259366
BH	MOL21	26-O-beta-D-Glucopyranosyl-3Beta,26-dihydroxy-choleslen-16,22-dioxo-3-O-Alpha-L-rhamnopyranosyl-(1-2)-beta-D-glucopyranoside_Qt	432.71	32.43	0.8	1	0	0.27559055
BH	MOL24	26-O-Β-D-Glucopyranosyl-3Β, 26-dihydroxy-cholestan-16, 22-dioxo-3-O-Α-L-rhamnopyranosyl-(1⟶2)-Β-D-glucopyranoside_Qt	432.71	32.43	0.8	1	0	0.27559055

**Table 3:** Core targets and topological parameters of BHT of antidepressant effect.

Target name	Protein name	BC	CC	Degree
AKT1	RAC-alpha serine/threonine-protein kinase	0.09042456	0.56896552	29
MAPK1	Mitogen-activated protein kinase 1	0.11614601	0.58928571	28
VEGFA	Vascular endothelial growth factor A	0.04736493	0.54545455	24
INS	Insulin	0.08146496	0.55	23
TNF	Tumor necrosis factor	0.06669936	0.52380952	22
APP	Amyloid-beta A4 protein	0.14143673	0.53658537	21
NOS3	Nitric oxide synthase, endothelial	0.01819553	0.51162791	17
BDNF	Brain-derived neurotrophic factor	0.10551453	0.52380952	17
HRAS	GTPase HRas	0.04286645	0.50381679	16
HSP90AA1	Heat shock protein HSP 90-alpha	0.0302578	0.47826087	15
CASP3	Caspase-3	0.02845487	0.50381679	15
PTGS2	Prostaglandin G/H synthase 2	0.02909971	0.47482014	14

**Table 4:** Linear regression equation of components.

Neurotransmitter	Regression equation	*R*	Linear range (*μ*g/ml)
DA	*Y* = 3008.5*X* − 70.842	0.9954	0.001∼1.0
NE	*Y* = 10.484*X* − 0.9794	0.9981	0.001∼1.0
5-HT	*Y* = 74.796*X* − 0.7164	0.9952	0.001∼1.0
Glu	*Y* = 468.93*X* − 69.387	0.9999	0.5∼50.0

**Table 5:** UPLC-Q-TOF-MS analysis results of BHT.

RT(min)	Ionization mode	(*m*/*z*)	Daughter fragment ion	Identified compounds
2.26	ESI-	415.1298	179.0361, 161.0268	Regaloside C [24], identification by reference
3.55	ESI-	399.1323	163.0412, 145.0315	Regaloside A [24, 25], identification by reference
4.34	ESI-	429.1370	193.0526, 175.0432	Regaloside F [24]
4.65	ESI-	237.0798	163.0465, 119.0516	1-*o*-*p*-Coumaroylglycerol [24]
5.69	ESI-	457.1384	397.1159, 161.0242	Regaloside E [24], identification by reference
7.76	ESI-	441.1436	381.1205, 163.0412	Regaloside B [24, 25], identification by reference
16.51	ESI-	413.1317	193.0555, 163.0465	1-*o*-Feruloyl-3-*o*-*p*-coumaroylglycerol [24]

**Table 6:** Content of characteristic components of BHT.

No.	Batch	Regaloside A (mg/ml)	Regaloside B (mg/ml)	Regaloside C (mg/ml)	Regaloside E (mg/ml)
S1	Longshan 20190612	13.76	9.15	0.57	0.78
S2	Longshan 20190522	12.99	8.26	0.65	0.71
S3	Longshan 20190418	13.89	8.54	0.69	0.82
S4	Enshi 20190521	12.14	8.02	0.67	0.75
S5	Enshi 20190502	11.05	4.68	0.41	0.60
S6	Enshi 20190622	12.84	9.41	0.34	0.87

## Data Availability

The data used to support the findings of this study are included within the article.
